# Pretreatment Prognostic Nutritional Index Is Associated with Treatment Response and Survival in Patients with Relapsed or Refractory Non-Hodgkin Lymphoma Receiving CAR-T Cell Therapy

**DOI:** 10.3390/nu18203425

**Published:** 2026-10-19

**Authors:** Zhengyuan Han, Yadi Zhong, Zijian Lv, Weidong Han, Yang Liu

**Affiliations:** 1 Department of Bio-Therapeutic, The First Medical Center, Chinese PLA General Hospital, Beijing 100853, China; hanzhengyuan6@163.com (Z.H.); zhongyadi101@sina.com (Y.Z.); lvzijian99@163.com (Z.L.); 2 Department of Hematology, The Second Medical Center & National Clinical Research Center for Geriatric Diseases, Chinese PLA General Hospital, Beijing 100853, China

**Keywords:** prognostic nutritional index, PNI, CAR-T therapy, TanCAR7, relapsed/refractory B-cell non-Hodgkin lymphoma, nutrition, survival, risk stratification

## Abstract

**Background**: Clinical outcomes after CAR-T therapy vary substantially in patients with relapsed or refractory B-cell non-Hodgkin lymphoma (r/r B-NHL), and practical markers available before infusion are still limited. The prognostic nutritional index (PNI), calculated from serum albumin and absolute lymphocyte count, reflects both nutritional and immune condition. Whether pretreatment PNI has prognostic value in patients treated with TanCAR7 remains unclear. **Methods**: We performed a retrospective analysis of patients with r/r B-NHL who received TanCAR7, a tandem CD19/CD20-directed CAR-T product, in a single-center phase I/II trial. Pretreatment PNI was assessed using routine laboratory data obtained before lymphodepletion and infusion. Associations between PNI and treatment response, progression-free survival (PFS), and overall survival (OS) were analyzed. Survival outcomes were evaluated with Kaplan–Meier methods and Cox regression models. **Results**: A total of 64 patients were included in the final analysis, including 27 patients in the low-PNI group and 37 in the high-PNI group. Patients with high pretreatment PNI had a significantly higher complete response rate (81.1% vs. 51.9%, *p* = 0.021) and objective response rate (89.2% vs. 63.0%, *p* = 0.024) than those with low pretreatment PNI. Kaplan–Meier analysis showed that high pretreatment PNI was associated with longer PFS (*p* = 0.006) and OS (*p* = 0.012). In multivariable analysis, high pretreatment PNI remained associated with improved PFS (HR = 0.202, 95% CI 0.076–0.538, *p* = 0.001) and OS (HR = 0.217, 95% CI 0.084–0.564, *p* = 0.002), although these estimates should be interpreted cautiously given the limited number of events and the apparent time-varying effect of PNI. Time-dependent ROC analysis showed that pretreatment PNI had better discriminative performance for shorter-term outcomes, particularly within the first 1–2 years after infusion. **Conclusions**: In patients with r/r B-NHL treated with TanCAR7, pretreatment PNI was associated with treatment response and survival. As a simple index derived from routine laboratory tests, PNI may help refine pretreatment risk assessment and identify patients who may benefit from closer early monitoring and supportive care. Prospective validation in larger cohorts is needed.

## 1. Introduction

B-cell non-Hodgkin lymphoma (B-NHL) comprises a heterogeneous group of mature lymphoid malignancies with marked variation in pathology, clinical presentation, and prognosis across subtypes [1,2,3,4,5,6,7]. Lymphoma remains an important contributor to the global cancer burden. GLOBOCAN 2020 estimated more than 540,000 new cases and about 260,000 deaths from non-Hodgkin lymphoma worldwide, underscoring its public health relevance [2]. Recent cancer statistics from the United States likewise confirm the persistent burden of lymphoma incidence and mortality [3]. Although many patients benefit from modern first-line immunochemotherapy, relapse or treatment resistance still develops in a substantial proportion of cases, and outcomes after failure of prior therapy remain unsatisfactory [8,9,10,11]. In these patients, therapeutic decision-making becomes increasingly difficult because recurrent disease is often accompanied by cumulative toxicity from previous treatment, greater tumor burden, and reduced physiologic reserve.

The introduction of chimeric antigen receptor T-cell (CAR-T) therapy has markedly expanded treatment options for relapsed or refractory B-cell lymphomas [12,13,14,15,16,17]. Durable remission can be achieved in some heavily pretreated patients, and clinical activity has also been shown in lymphoma subtypes beyond large B-cell lymphoma, including follicular lymphoma and mantle cell lymphoma [18,19,20,21,22]. Even so, treatment outcomes are highly variable. While some patients experience prolonged disease control, others derive only limited benefit or progress soon after infusion [16,23,24,25]. This heterogeneity indicates that prognosis after CAR-T therapy cannot be explained solely by tumor characteristics [26,27,28]. Patient-related factors before infusion are also likely to influence subsequent outcome [18,29,30,31,32,33].

This question is especially relevant in candidates for CAR-T therapy, who frequently reach this stage after multiple prior lines of treatment. By the time of referral, some patients have already developed impaired marrow reserve, ongoing inflammatory activation, immune dysfunction, or poor nutritional status [29,33]. These features may affect both tolerance to treatment and long-term outcome, yet they are not adequately captured by conventional lymphoma-focused prognostic factors. For this reason, there is a clear need for pretreatment markers that are practical, reproducible, and available in routine clinical care.

In general, pretreatment prognostic assessment is useful because it may help identify patients at higher risk, guide supportive management, and improve baseline risk stratification before intensive or complex therapies. The prognostic nutritional index (PNI) is a simple measure derived from serum albumin concentration and peripheral lymphocyte count, and it provides indirect information on nutritional condition as well as host immune status [34,35,36,37]. In several solid tumors and hematologic malignancies, a lower PNI has been linked to less favorable outcomes [34,35,38,39,40]. Evidence has also begun to emerge suggesting that immunonutritional markers may have prognostic value in patients receiving CAR-T therapy [41,42,43]. Given that albumin and lymphocyte count may together reflect systemic inflammation, nutritional reserve, and immune competence, pretreatment PNI may be a clinically useful marker in this setting.

TanCAR7 is a tandem CD19/CD20-targeted CAR-T product developed to reduce the limitations of single-antigen targeting, particularly relapse associated with antigen escape, and early clinical studies have shown promising activity in relapsed or refractory B-cell lymphoma [31,32,44,45,46]. However, baseline factors associated with efficacy and survival after TanCAR7 infusion have not been fully characterized. In particular, the prognostic relevance of pretreatment PNI in this population remains unclear.

Therefore, we performed a retrospective study in patients with relapsed or refractory B-NHL treated with TanCAR7 to evaluate whether pretreatment PNI was associated with treatment response, progression-free survival (PFS), and overall survival (OS). We also explored its potential role as a simple baseline marker for risk stratification before CAR-T therapy.

## 2. Materials and Methods

### 2.1. Study Design and Reporting Framework

This study was conducted as a retrospective observational analysis of a prospectively treated cohort and is reported in line with the Strengthening the Reporting of Observational Studies in Epidemiology (STROBE) recommendations [47,48]. The analysis was based on patients enrolled in a single-center phase I/II study of TanCAR7 at the Chinese PLA General Hospital (ClinicalTrials.gov: NCT03097770).

TanCAR7 is a dual-target CAR-T product directed against CD19 and CD20 and was developed for patients with relapsed or refractory B-cell non-Hodgkin lymphoma. The current study was not intended to re-report the parent trial outcomes; rather, its purpose was to explore whether pretreatment nutritional and immune status, as reflected by the prognostic nutritional index (PNI), was related to subsequent response and survival after TanCAR7 infusion.

**Figure 1:**
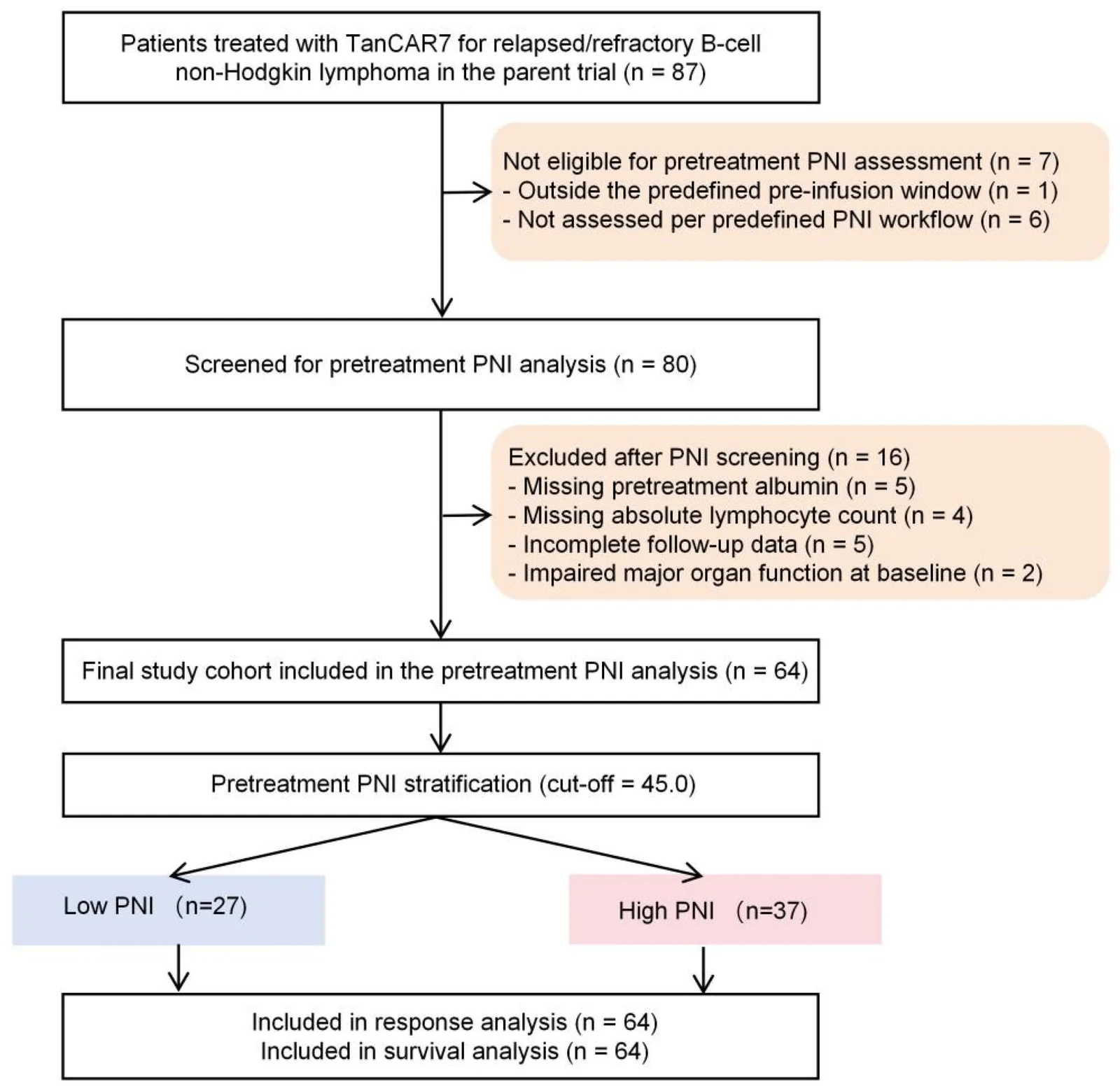
Flowchart of patient selection for the pretreatment PNI analysis. Reasons for exclusion before and after PNI screening are shown.

Of the 87 patients who underwent TanCAR7 infusion in the parent trial, 80 were considered eligible for assessment in the present study. Sixteen were excluded because the variables required to calculate pretreatment PNI were unavailable, follow-up information was insufficient, or key data needed for the planned analyses were missing. The final study set included 64 patients. Details of patient selection are provided in Figure 1.

Some patients included here were also part of the previously published long-term follow-up analysis of the TanCAR7 trial. However, the present work addressed a separate biomarker-oriented question and used a different analytic framework.

### 2.2. Treatment and Follow-Up

Before cell infusion, all patients received lymphodepleting chemotherapy according to the trial protocol. The administered TanCAR7 dose ranged from 0.5 × 10^6^ to 10.0 × 10^6^ CAR-T cells/kg. Additional supportive treatment, including bridging approaches when needed, was provided on clinical grounds.

Tumor response was evaluated at protocol-specified time points after infusion using imaging studies together with clinical assessment. Safety monitoring was performed within the parent study, with particular attention to cytokine release syndrome, neurologic complications, hematologic adverse events, and infections during the scheduled follow-up period. Longer-term surveillance also included delayed complications such as autoimmune conditions and second malignancies. Survival information continued to be updated even after documented disease progression.

### 2.3. Data Collection and Sources

Data for this study were obtained from two sources: the prospective TanCAR7 trial database and the corresponding hospital medical records. Baseline information extracted for analysis included demographic features, performance status, histologic diagnosis, disease burden-related characteristics, prior treatment exposure, bridging therapy, laboratory findings, and infused CAR-T cell dose. Data on response, survival, and treatment-related toxicities were collected from scheduled trial assessments and follow-up records.

Laboratory values used for pretreatment PNI calculation were taken from routine blood tests performed before lymphodepletion and before TanCAR7 infusion. Only patients with sufficient pretreatment data and analyzable follow-up were retained in the final cohort.

### 2.4. Pretreatment Prognostic Nutritional Index and Baseline Variables

The prognostic nutritional index (PNI) was calculated from the last available pre-infusion laboratory measurements. In most patients, these samples were obtained within 3 to 7 days before TanCAR7 infusion and before the start of lymphodepleting chemotherapy. PNI was defined as follows:

PNI = serum albumin (g/L) + 5 × absolute lymphocyte count (×10^9^/L)

For the main analysis, patients were assigned to groups using a cutoff of 45.0, selected a priori on the basis of previous reports rather than derived from the current dataset. To describe the performance of continuous PNI in this cohort, we also carried out a receiver operating characteristic analysis for objective response. The corresponding cohort-based optimal cutoff was presented for reference only and was not used to redefine the study groups.

Accordingly, patients were classified as having low PNI (<45.0) or high PNI (≥45.0); patients with a PNI value exactly equal to 45.0 were assigned to the high-PNI group. In the exploratory three-category analysis, pretreatment PNI was categorized as <40.0, 40.0 to <45.0, and ≥45.0; patients with a PNI value exactly equal to 40.0 were assigned to the middle category, whereas those with a value exactly equal to 45.0 were assigned to the highest category.

The baseline factors examined in this study included age, sex, Eastern Cooperative Oncology Group performance status, histologic subtype, Ann Arbor stage, lactate dehydrogenase level, prior lines of treatment, previous transplantation, bridging therapy, and CAR-T cell dose.

### 2.5. Outcomes

The parent trial evaluated treatment response, survival, and long-term safety after TanCAR7 therapy. In the present analysis, the efficacy outcomes of interest were best overall response, objective response rate (ORR), progression-free survival (PFS), and overall survival (OS).

Best overall response was categorized as complete response, partial response, stable disease, or progressive disease. ORR was defined as the proportion of patients who achieved either complete or partial response. Disease control rate was defined as the proportion of patients who achieved complete response, partial response, or stable disease.

PFS was measured from the date of TanCAR7 infusion to the first occurrence of disease progression or death from any cause. OS was measured from infusion to death from any cause. Patients who were alive and event-free at the time of last contact were censored on that date.

Safety analyses were exploratory and were limited to adverse events for which sufficiently defined data were available in the retrospective dataset. In particular, the relationship between pretreatment PNI group and the occurrence of cytokine release syndrome was examined. ICANS was not analyzed because standardized ICANS data were not available in the original dataset.

### 2.6. Statistical Analysis

Categorical variables are reported as counts and percentages, whereas continuous variables are summarized as medians with interquartile ranges. Comparisons between the low- and high-PNI groups were made using Fisher’s exact test for categorical data and the Mann–Whitney U test for continuous data.

The principal grouped analyses were based on the prespecified PNI threshold of 45.0, with an additional exploratory three-category analysis also performed as defined above. For descriptive purposes, receiver operating characteristic analysis was additionally used to examine the ability of continuous PNI to distinguish objective responders (CR or PR) from non-responders, with objective response coded as the positive outcome. The area under the curve together with the Youden-based optimal cutoff was recorded. Sensitivity and specificity at the prespecified cutoff of 45.0 were also presented for reference.

Time-to-event outcomes were analyzed with the Kaplan–Meier method, and differences between groups were assessed using the log-rank test. The effects of baseline factors on PFS and OS were estimated using Cox regression models, with results expressed as hazard ratios and 95% confidence intervals. Variables showing an association at *p* < 0.10 in univariable analysis, together with factors considered clinically important, were considered for multivariable analysis. Because the number of outcome events was limited, model construction was intentionally conservative. The main adjusted model included age, performance status, histology, lactate dehydrogenase, and PNI. Variables closely related to the components of PNI were not entered together with PNI in the same model.

As the study population included more than one histologic subtype of B-cell lymphoma, exploratory subgroup analyses were also performed to examine whether the association between PNI and outcome was consistent in the aggressive lymphoma subgroup.

The proportional hazards assumption for the multivariable Cox models was assessed using Schoenfeld residual-based tests for each covariate together with the corresponding global test, and residual plots were visually inspected. Because evidence of non-proportionality was observed for pretreatment PNI, an additional sensitivity analysis restricted to the first 24 months of follow-up was performed to examine whether the association between pretreatment PNI and survival was concentrated in the earlier post-infusion period. This restricted follow-up analysis was intended as a descriptive sensitivity analysis rather than a formal time-varying coefficient model.

To evaluate prognostic discrimination over time, time-dependent ROC analysis was carried out with continuous PNI treated as the marker of interest. A cumulative/dynamic framework was used, and censoring was handled by inverse probability of censoring weighting. Time-specific AUCs with 95% confidence intervals were estimated at 1, 2, 3, 4, and 5 years for both PFS and OS. Internal optimism was assessed by bootstrap resampling (1000 iterations), and both apparent and optimism-corrected AUC values were reported.

For exploratory safety comparisons, odds ratios with 95% confidence intervals were calculated where appropriate. All statistical tests were two-sided, and a *p* value < 0.05 was considered statistically significant. Analyses were performed using R software, version 4.3.0.

### 2.7. Ethical Considerations

The original TanCAR7 trial and the present secondary analysis were approved by the Medical Ethics Committee of the Chinese PLA General Hospital (approval No. S2017-049-01). The study was conducted in accordance with the Declaration of Helsinki. Written informed consent had been obtained from all participants before treatment in the parent trial.

## 3. Results

### 3.1. Patient Selection and Determination of the Pretreatment PNI Cutoff

The patient selection process is outlined in Figure 1. Among 87 patients with relapsed/refractory B-cell non-Hodgkin lymphoma treated with TanCAR7 in the parent trial, 7 were not eligible for the predefined pretreatment PNI assessment. Of these, 1 patient was outside the predefined pre-infusion assessment window and 6 were not evaluated according to the prespecified pretreatment PNI workflow. The remaining 80 patients were screened for inclusion in the present analysis. After screening, 16 patients were excluded because of missing pretreatment albumin data (*n* = 5), missing absolute lymphocyte count (*n* = 4), incomplete follow-up data (*n* = 5), or impaired major organ function at baseline (*n* = 2). The final study cohort therefore comprised 64 patients. To explore the possibility of selection bias, baseline characteristics were compared descriptively between the 64 included patients and the 23 excluded patients (Supplementary Table S1). The available comparisons did not identify obvious differences in age, sex, lymphoma aggressiveness, primary refractory disease, or tumor burden; however, these non-significant findings should be interpreted cautiously, as they do not establish equivalence between included and excluded patients and cannot exclude the possibility of selection bias.

**Figure 2:**
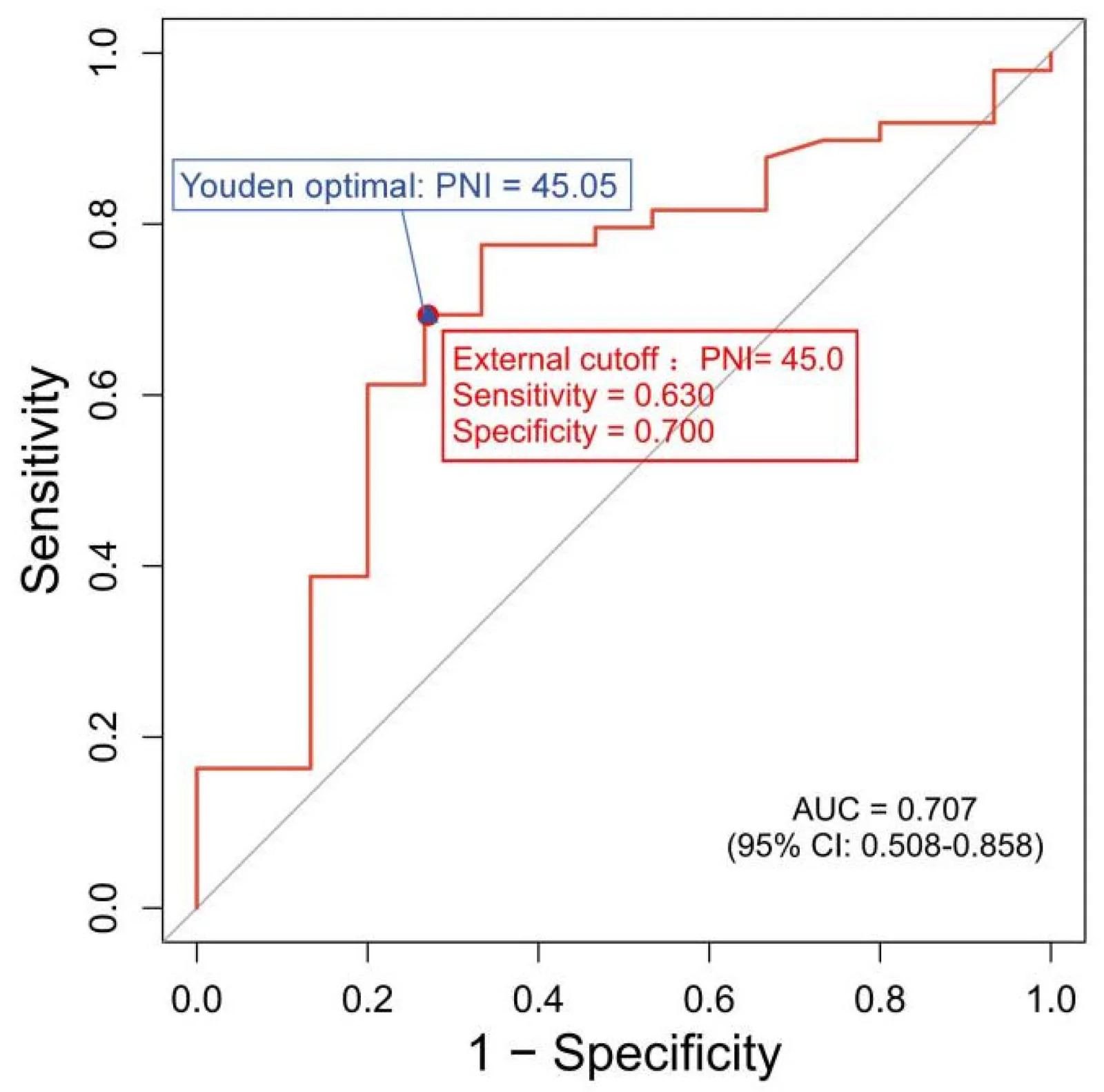

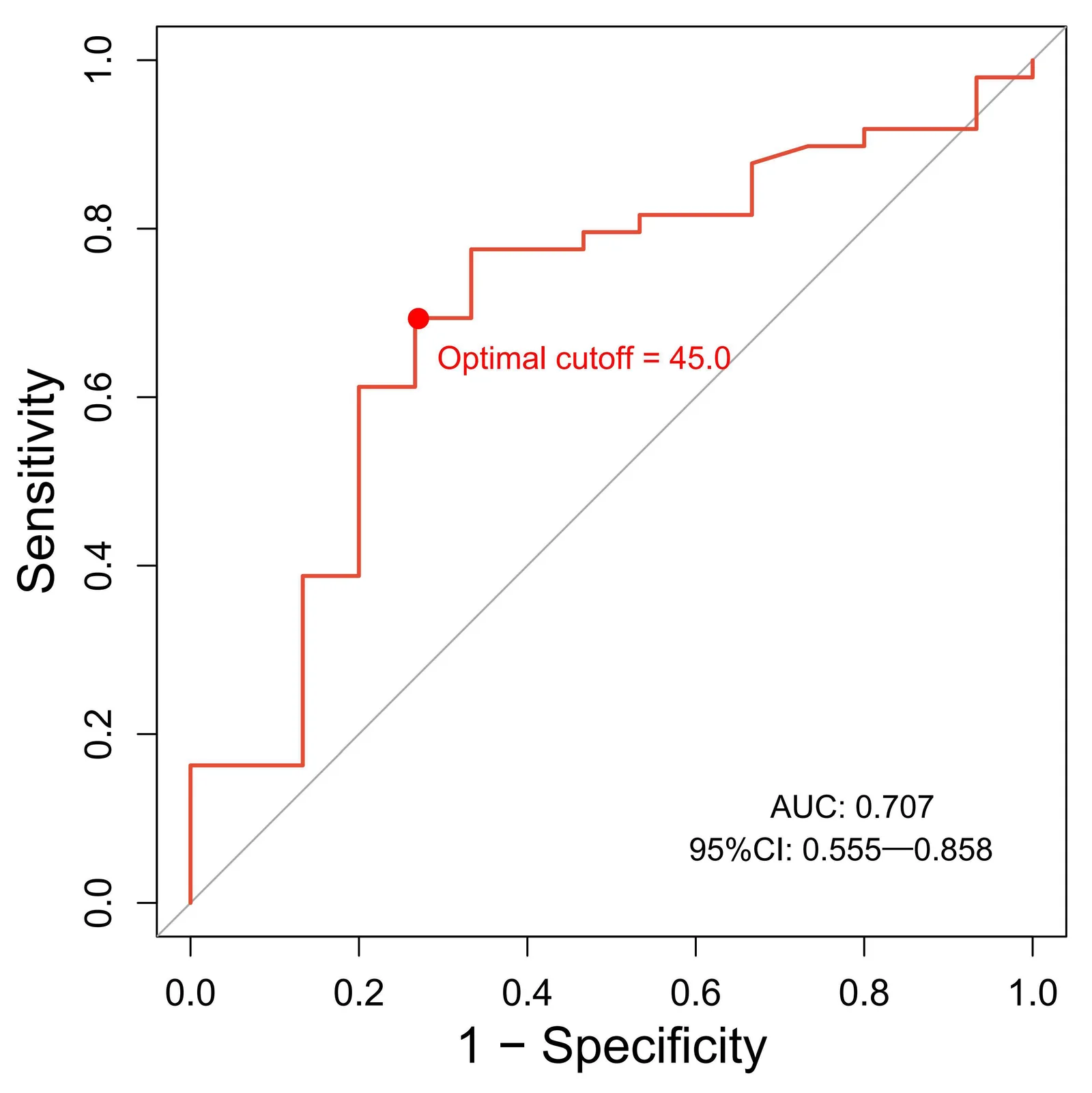
Receiver operating characteristic analysis of pretreatment prognostic nutritional index for objective response (CR/PR vs. non-response). The Youden-based optimal cutoff was 45.05. The prespecified cutoff of 45.0 is also shown for reference together with its corresponding sensitivity and specificity.

Pretreatment PNI was calculated as serum albumin (g/L) + 5 × absolute lymphocyte count (×10^9^/L). Using the prespecified threshold of 45.0, 27 patients were assigned to the low-PNI group and 37 to the high-PNI group. We then examined whether this threshold was supported by the current dataset. In receiver operating characteristic analysis for objective response, with responders defined as the positive outcome, the area under the curve was 0.707 (95% CI, 0.508–0.858). The Youden index identified an optimal cutoff of 45.05. For reference, the prespecified cutoff of 45.0 yielded a sensitivity of 0.630 and a specificity of 0.700, as shown in Figure 2. Because this data-derived cutoff was almost identical to the predefined value, the threshold of 45.0 was retained for all subsequent analyses.

### 3.2. Baseline Characteristics According to Pretreatment PNI Group

Baseline characteristics of the 64 included patients are summarized in Table 1. Overall, 10 patients (15.6%) were older than 60 years, 29 (45.3%) were male, and 42 (65.6%) had diffuse large B-cell lymphoma. Most patients had aggressive lymphoma (79.7%), primary refractory disease (60.9%), and advanced-stage disease (82.8%).

When patients were stratified by pretreatment PNI, several differences became apparent. Compared with the high-PNI group, those in the low-PNI group more often had an IPI score of 3 or higher (59.3% vs. 29.7%, *p* = 0.023) and a greater tumor burden as assessed by SPD ≥ 39.5 cm^2^ (70.4% vs. 35.1%, *p* = 0.011). As expected, low PNI was also strongly associated with lower serum albumin before CAR-T infusion, with albumin < 40.5 g/L observed in 85.2% of patients in the low-PNI group compared with 24.3% in the high-PNI group (*p* < 0.001).

### 3.3. Treatment Response According to Pretreatment PNI

Best overall response according to pretreatment PNI group is summarized in Table 2. In the overall cohort, 44 of 64 patients (68.8%) achieved complete response (CR), 6 (9.4%) achieved partial response (PR), 4 (6.3%) had stable disease (SD), and 10 (15.6%) experienced progressive disease (PD). The objective response rate (ORR) and disease control rate (DCR) for the entire cohort were 78.1% and 84.4%, respectively.

Patients with high pretreatment PNI showed a more favorable response profile than those with low pretreatment PNI. The CR rate was significantly higher in the high-PNI group than in the low-PNI group (81.1% vs. 51.9%, *p* = 0.021). Likewise, ORR was higher in patients with high PNI (89.2% vs. 63.0%, *p* = 0.024). Although PD was numerically less frequent in the high-PNI group (8.1% vs. 25.9%) and DCR was also higher (91.9% vs. 74.1%), these differences did not reach statistical significance.

To further explore the clinical interpretation of pretreatment PNI categories, we performed an exploratory analysis using three PNI strata (Supplementary Table S2). Patients with PNI ≥ 45 had the most favorable treatment response and survival outcomes, with a CR rate of 81.1%, an ORR of 89.2%, and 1-year and 2-year OS rates of 100.0% and 97.3%, respectively. The PNI 40 to <45 subgroup showed lower response rates than the PNI < 40 subgroup (CR: 50.0% vs. 66.7%; ORR: 66.7% vs. 76.2%), indicating that the response pattern across the three categories was not strictly monotonic. Median PFS and OS were shortest in the PNI 40 to <45 subgroup, whereas both were not reached in the PNI ≥ 45 subgroup. Given the small number of patients in the intermediate subgroup, these findings should be interpreted cautiously, but they remained broadly consistent with the main dichotomous analysis showing more favorable outcomes in patients with higher pretreatment PNI. Because the study cohort included both aggressive and indolent B-cell lymphomas, an additional subgroup analysis was performed by lymphoma subtype (Supplementary Table S3). In the aggressive lymphoma subgroup, high pretreatment PNI remained associated with a higher CR rate (86.7% vs. 57.1%, *p* = 0.024), while the difference in ORR showed the same direction but did not reach statistical significance (90.0% vs. 66.7%, *p* = 0.064). In contrast, no clear difference in response was observed within the indolent lymphoma subgroup, although the sample size in that subgroup was limited.

### 3.4. Survival Outcomes According to Pretreatment PNI

**Figure 3:**
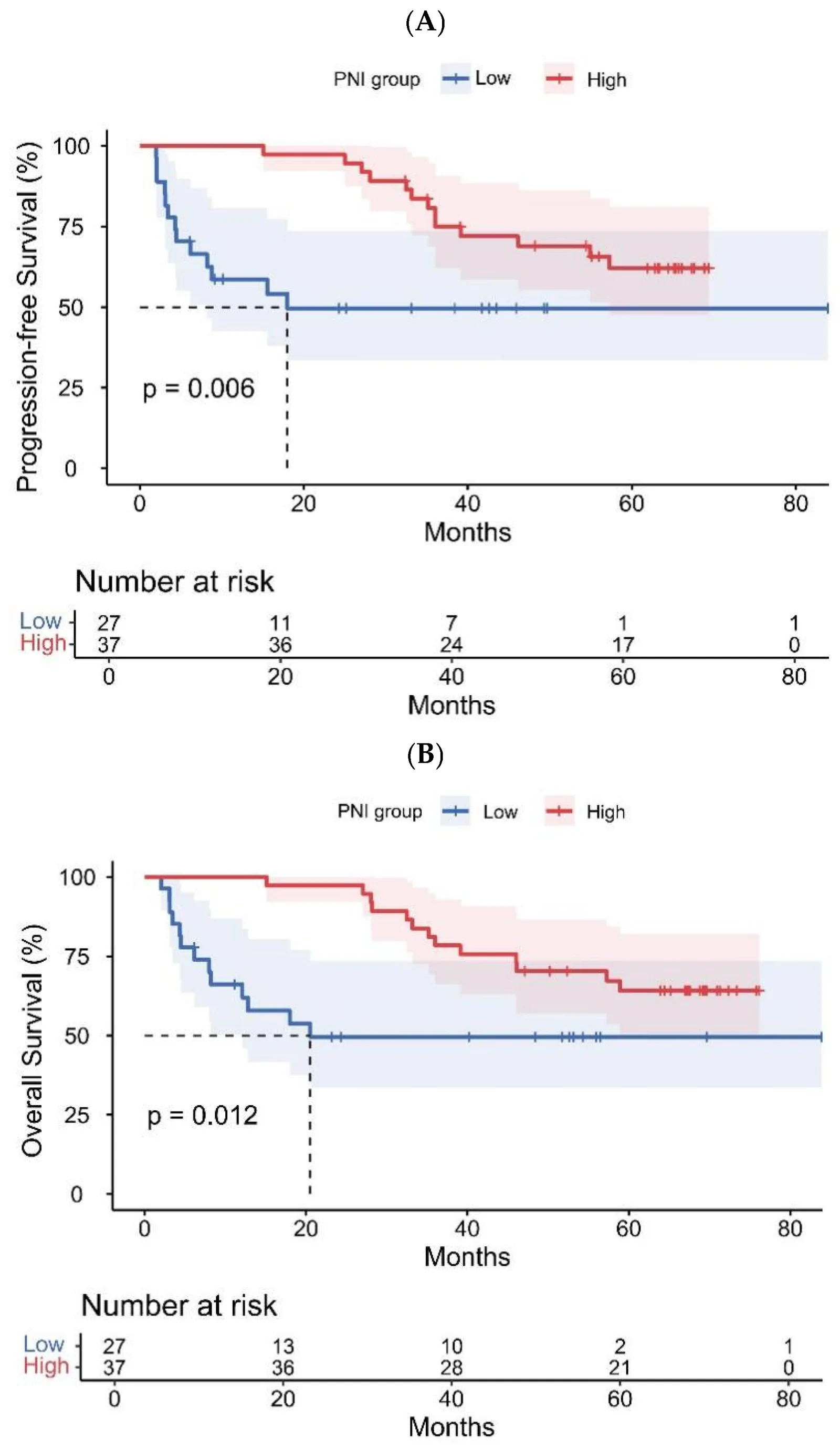
(**A**,**B**) Kaplan–Meier survival curves for progression-free survival (PFS) and overall survival (OS) stratified by PNI group.

Survival outcomes according to pretreatment PNI group are shown in Figure 3. Patients with high pretreatment PNI had significantly better progression-free survival (PFS) and overall survival (OS) than those with low pretreatment PNI. The separation between the Kaplan–Meier curves was evident early after CAR-T infusion and remained apparent during follow-up. Log-rank testing showed significant differences for both PFS (*p* = 0.006) and OS (*p* = 0.012).

To further describe the temporal pattern of mortality, cumulative deaths at prespecified follow-up time points were examined descriptively (Table 3). These cumulative death counts are presented as descriptive summaries only and do not replace time-to-event estimates that account for censoring, such as the Kaplan–Meier and Cox regression analyses. During the first year, all 9 recorded deaths occurred in the low-PNI group, whereas no deaths were observed in the high-PNI group (*p* = 0.002). By 2 years, cumulative deaths remained markedly higher among patients with low pretreatment PNI (13 vs. 1, *p* < 0.001). This difference persisted at 3 years (13 vs. 8, *p* = 0.033), but by 5 years the cumulative number of deaths was identical in the two groups (13 vs. 13, *p* = 0.316).

Taken together, these findings indicate that pretreatment PNI was associated with survival after CAR-T therapy, with the prognostic separation being most pronounced during the earlier phase of follow-up.

### 3.5. Discriminative Performance and Cox Regression Analyses for Survival

**Figure 4:**
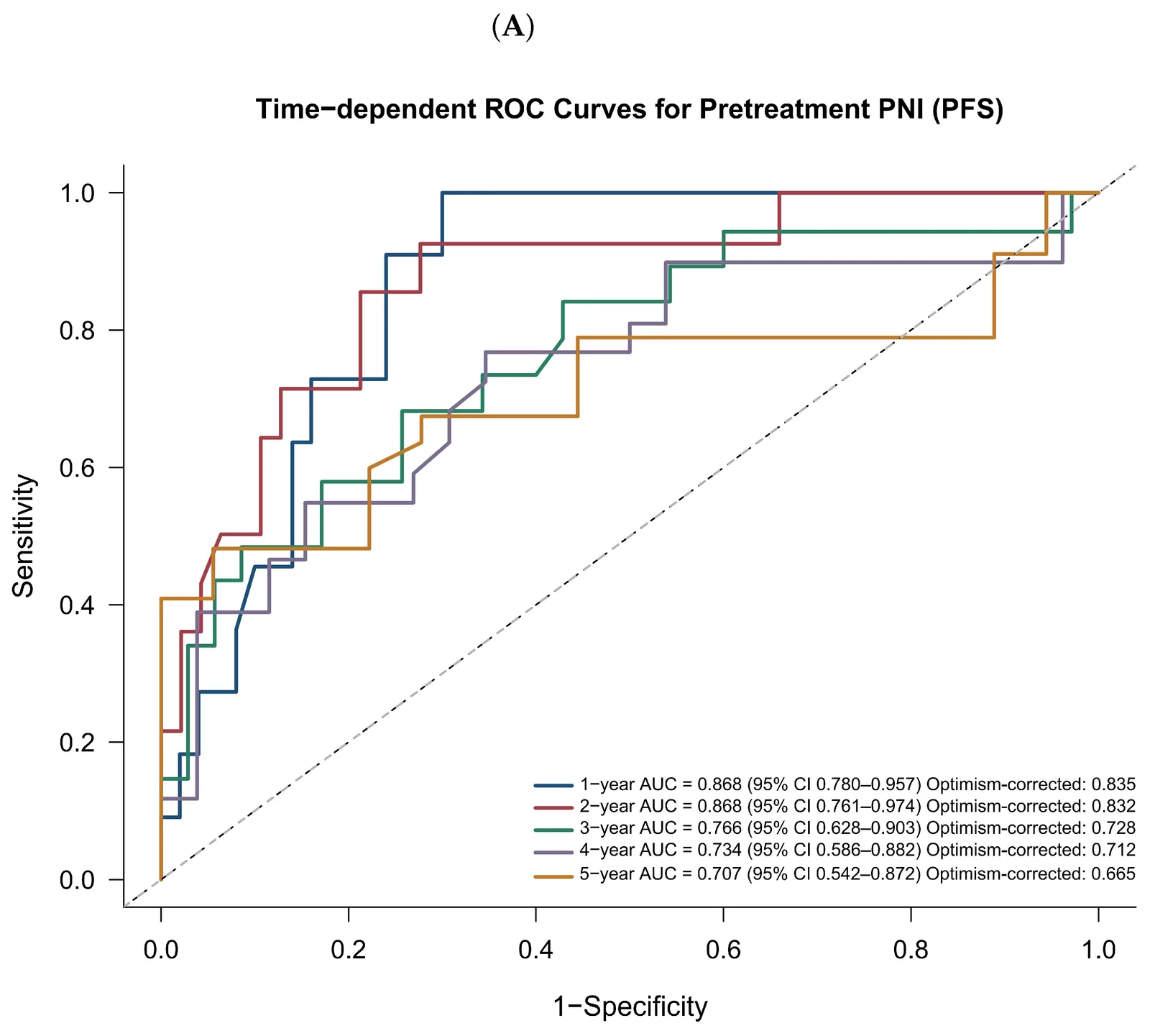

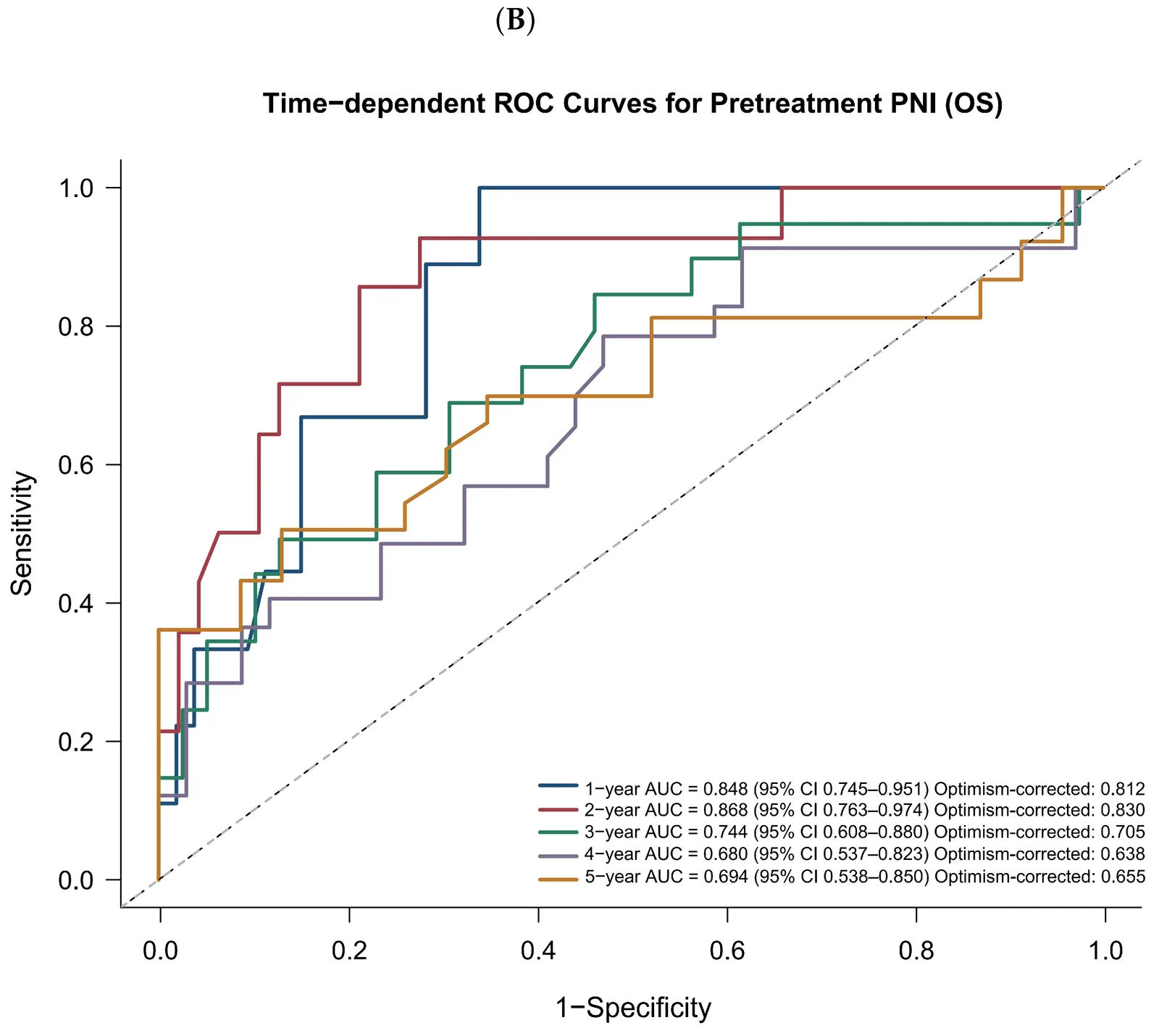
Time-dependent receiver operating characteristic analysis of pretreatment prognostic nutritional index for survival outcomes. (**A**) Progression-free survival. (**B**) Overall survival.

The prognostic performance of continuous pretreatment PNI was further assessed using time-dependent ROC analysis for PFS and OS (Figure 4A,B). For PFS, the apparent AUCs at 1, 2, 3, 4, and 5 years were 0.868, 0.868, 0.766, 0.734, and 0.707, respectively; the corresponding optimism-corrected AUCs were 0.835, 0.832, 0.728, 0.712, and 0.665. For OS, the apparent AUCs at the same time points were 0.848, 0.868, 0.744, 0.680, and 0.694, with optimism-corrected AUCs of 0.812, 0.830, 0.705, 0.638, and 0.655, respectively. Overall, PNI showed better discriminative ability for short-term outcomes, particularly within the first 1–2 years after infusion.

**Figure 5:**
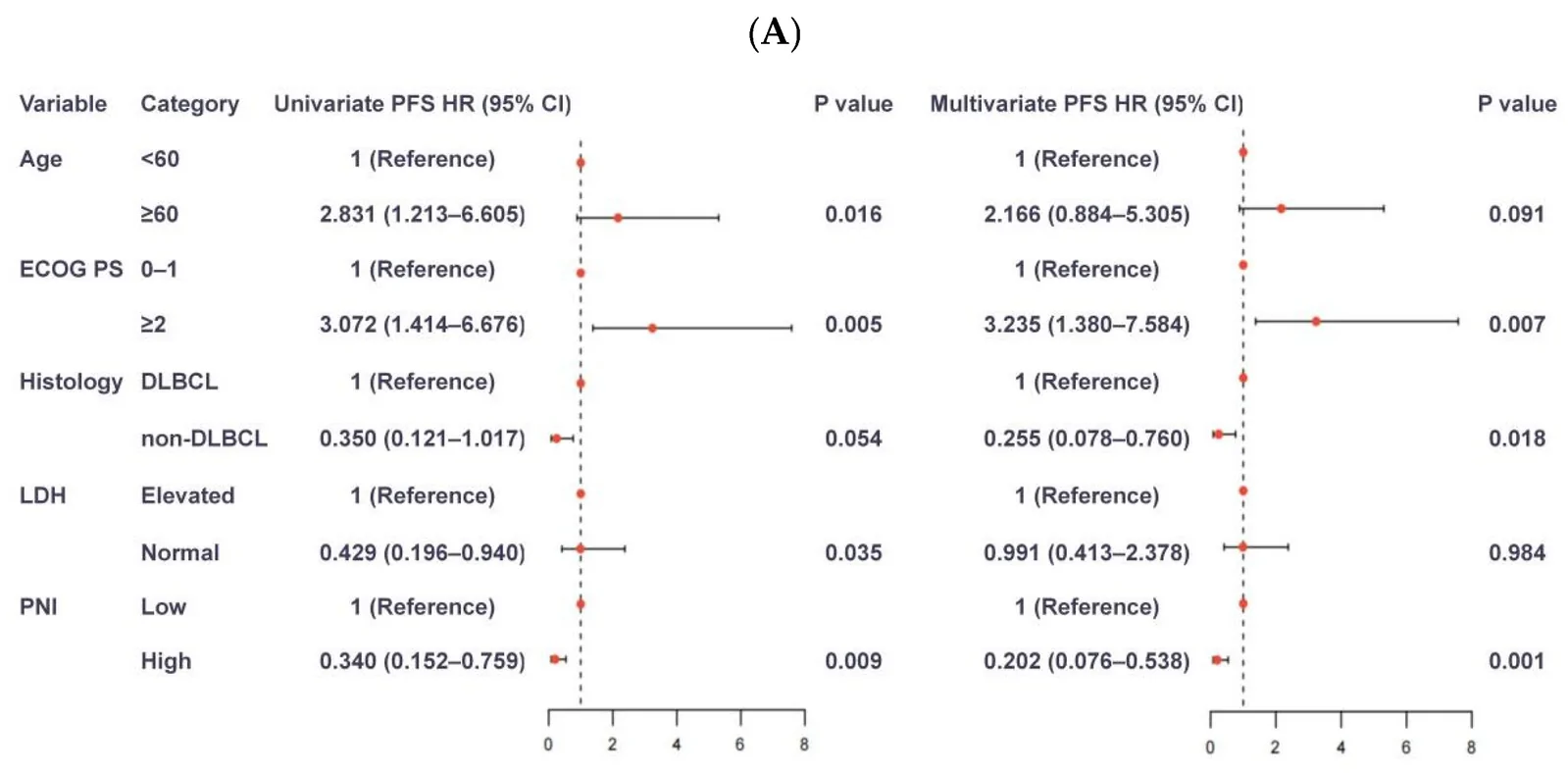

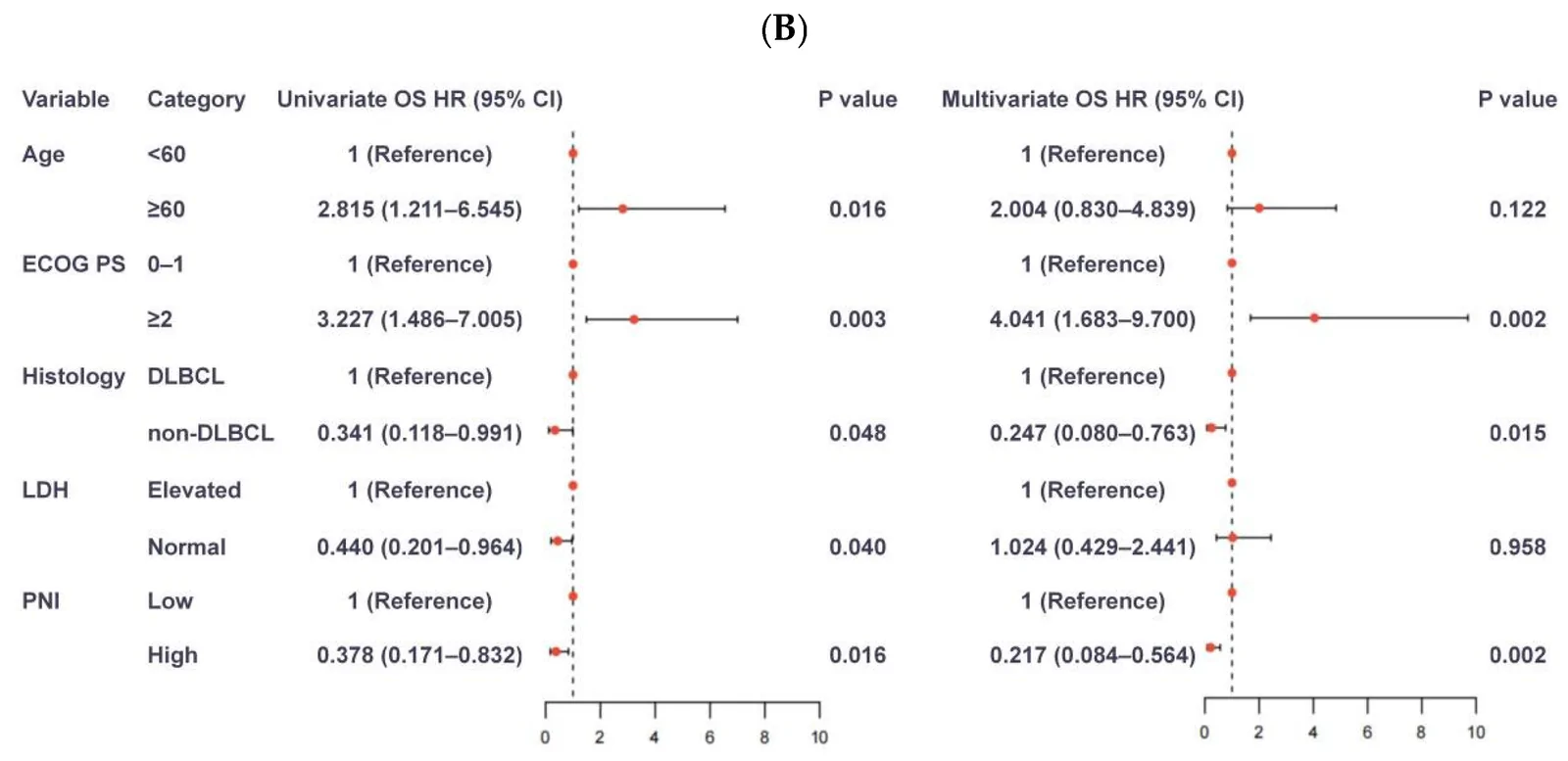
Univariable and multivariable Cox regression analyses for survival outcomes. (**A**) Progression-free survival. (**B**) Overall survival.

The results of the Cox regression analyses are shown in Figure 5. In univariable analysis for PFS, age ≥60 years, ECOG performance status of 2, elevated LDH, and low pretreatment PNI were associated with worse outcomes, whereas non-DLBCL histology showed a borderline association. In the multivariable model, ECOG performance status of ≥2 (HR = 3.235, 95% CI, 1.380–7.584, *p* = 0.007), non-DLBCL histology (HR = 0.255, 95% CI, 0.078–0.760, *p* = 0.018), and high pretreatment PNI (HR = 0.202, 95% CI, 0.076–0.538, *p* = 0.001) remained associated with PFS after adjustment for the selected baseline covariates; however, these adjusted estimates should be interpreted cautiously given the limited number of events and the subsequent evidence that the effect of PNI was not constant over time. For OS, univariable analysis identified age ≥60 years, ECOG performance status of 2, elevated LDH, DLBCL histology, and low pretreatment PNI as adverse factors. In the multivariable analysis, ECOG performance status of 2 (HR = 4.041, 95% CI, 1.683–9.700, *p* = 0.002), non-DLBCL histology (HR = 0.247, 95% CI, 0.080–0.763, *p* = 0.015), and high pretreatment PNI (HR = 0.217, 95% CI, 0.084–0.564, *p* = 0.002) remained significantly associated with OS after adjustment for the selected baseline covariates.

Because the Kaplan–Meier curves separated mainly during the early phase of follow-up, we further assessed the proportional hazards assumption. Schoenfeld residual-based testing showed significant global departures from proportional hazards in both the multivariable PFS model (global test *p* = 0.047) and the multivariable OS model (global test *p* = 0.022). Among the individual covariates, pretreatment PNI showed clear evidence of a time-varying effect in both models (PFS: ρ = 0.666, *p* = 0.001; OS: ρ = 0.720, *p* < 0.001), whereas age (>60 years), ECOG performance status (≥2), histology, and elevated LDH did not show significant violations of the proportional hazards assumption. Visual inspection of the Schoenfeld residual plots for pretreatment PNI also supported a clear time-dependent pattern (Supplementary Figure S1).

To further explore the clinical implications of this finding, we performed a sensitivity analysis restricted to the first 24 months of follow-up (Supplementary Table S4). Within this early follow-up window, low pretreatment PNI was associated with substantially increased risks of progression (HR, 25.76; 95% CI, 3.35–197.83; *p* = 0.002) and death (HR, 25.64; 95% CI, 3.34–196.73; *p* = 0.002); however, these estimates were derived from a limited number of early events and should be viewed as exploratory. This restricted follow-up analysis was used to examine whether the prognostic association of pretreatment PNI was concentrated in the early post-infusion period; however, it should not be interpreted as a formal model of time-varying coefficients.

### 3.6. Exploratory Safety and Subgroup Analyses

An exploratory analysis of treatment-related toxicity according to pretreatment PNI is presented in Supplementary Table S4. Overall, any-grade cytokine release syndrome (CRS) occurred in 56 of 64 patients (87.5%). Although the incidence of any-grade CRS was numerically higher in the low-PNI group than in the high-PNI group (96.3% vs. 81.1%), this difference was not statistically significant (*p* = 0.124). In contrast, grade ≥2 CRS was more frequently observed in patients with low pretreatment PNI (29.6% vs. 8.1%; odds ratio, 4.77; 95% CI, 1.13–20.16; *p* = 0.042). A similar numerical trend was seen for grade ≥3 CRS (14.8% vs. 2.7%), although this association did not reach statistical significance (*p* = 0.153) (Table 4).

Given the histologic heterogeneity of the full cohort, an exploratory survival analysis was additionally performed in the aggressive lymphoma subgroup. As shown in the subgroup Kaplan–Meier curves (Supplementary Figure S2), patients with high pretreatment PNI continued to show superior survival outcomes, with significant differences in both PFS (*p* < 0.001) and OS (*p* = 0.002). These findings were consistent with the response and multivariable analyses, supporting the prognostic relevance of pretreatment PNI in patients with aggressive disease.

## 4. Discussion

Patients considered for CAR-T therapy often enter treatment after repeated lines of chemotherapy, immunotherapy, or transplantation, and their baseline condition is frequently shaped not only by lymphoma burden but also by cumulative treatment-related toxicity, chronic inflammation, lymphopenia, and reduced nutritional reserve. For this reason, we explored whether pretreatment PNI, a readily available index derived from albumin and lymphocyte count, could provide additional prognostic information in patients with relapsed or refractory B-cell non-Hodgkin lymphoma receiving TanCAR7.

The main finding of this study is that pretreatment PNI was associated with both treatment response and survival. Patients with lower PNI had less favorable response and experienced shorter PFS and OS, and this association remained evident after adjustment for selected baseline factors, although the adjusted results should be interpreted cautiously given the limited sample size and number of events. In addition, the prognostic effect of PNI appeared to be more apparent during the early period after infusion. This pattern suggests that pretreatment host condition may be especially relevant during the initial months after CAR-T therapy, when disease control, hematologic recovery, and treatment-related complications intersect [29,30,33]. The observed association is clinically plausible. PNI combines two simple measures that capture different aspects of patient status. Albumin is influenced by inflammation, catabolic state, organ function, and nutritional intake, whereas peripheral lymphocyte count reflects, at least in part, immune competence and prior treatment-related immune injury [34,35,36,37]. In a population as heavily pretreated as ours, a low PNI may therefore identify patients with limited physiologic reserve before infusion. Such patients may be less able to tolerate lymphodepletion, recover from prolonged cytopenias, or withstand infectious and metabolic stress during the early post-treatment course [23,29,30,49,50]. In this sense, PNI is unlikely to represent nutrition alone; rather, it may act as an accessible summary marker of host vulnerability.

Our findings are also consistent with the broader literature on immunonutritional markers in cancer. Lower PNI has been associated with adverse prognosis across several hematologic and solid malignancies [35,36,37,38,39,40,43]. More recently, related observations have begun to emerge in the setting of cellular immunotherapy. In patients with relapsed or refractory multiple myeloma treated with CAR-T therapy, baseline nutritional indices were reported to correlate with survival [41]. A recent study in non-Hodgkin lymphoma likewise suggested that a PNI-based immunonutritional model may help predict outcome after CD19 CAR-T therapy [42]. Although the specific indices and patient populations vary, these reports support the same general concept: outcome after CAR-T is influenced not only by tumor biology, but also by the condition of the host at the time treatment is delivered.

An important clinical issue is how pretreatment PNI should be interpreted in practice, especially given that the CAR-T treatment strategy itself was not different between patients with low and high PNI in this cohort. Based on the present findings, pretreatment PNI should not be considered a stand-alone basis for changing the CAR-T product, altering lymphodepletion, or withholding CAR-T therapy in otherwise eligible patients. At the current stage, its more realistic clinical value lies in baseline risk stratification and supportive care planning. In other words, once a patient’s PNI is known, the main implication is not necessarily to alter the antitumor protocol, but to refine how the patient is prepared for, monitored during, and supported after therapy.

For physicians, a low pretreatment PNI may justify a more detailed baseline review before infusion. This includes careful assessment of nutritional decline, occult infection, inflammatory burden, marrow reserve, and factors that may compromise recovery after treatment. It may also support more vigilant follow-up during the early post-infusion period, when adverse events and early progression are most likely to occur [49,50]. In practical terms, this could mean more frequent laboratory surveillance, closer observation for prolonged cytopenias, lower thresholds for infection workup, and earlier referral for nutritional or rehabilitation support when indicated [51,52,53]. Thus, while the core CAR-T regimen may remain unchanged, the intensity of supportive management may reasonably differ according to PNI-defined risk.

For patients, knowledge of a low PNI may help frame expectations more realistically. A low score should not be interpreted as a reason to abandon treatment, nor does it mean that response cannot be achieved. Rather, it indicates that the patient may require closer observation and more active supportive care. From the patient perspective, this may translate into earlier dietary counseling, more attention to weight loss or poor intake, prompt reporting of fever or functional decline, and closer adherence to follow-up visits after infusion [52,53]. Importantly, our study does not provide evidence that pretreatment PNI alone should lead to a different post-treatment medication regimen. Likewise, although nutritional intervention is reasonable in patients with low PNI, our data do not show that improving PNI before CAR-T will necessarily improve survival. That possibility remains hypothesis-generating and requires prospective study. At present, our data do not support a standardized or protocol-mandated change in post-treatment medications, dietary management, or lifestyle recommendations solely on the basis of pretreatment PNI.

The present findings should be interpreted in the context of several limitations. First, this was a retrospective single-center study with a relatively small sample size, and all patients were treated with the same CAR-T platform. At the same time, this cohort represents a highly selected TanCAR7 population, reflecting the stringent eligibility criteria and the early clinical experience with this novel dual-target CAR-T product. Notably, the main prognostic findings persisted even after restricting the cohort to aggressive large B-cell lymphomas, supporting the robustness of the results despite the histological diversity of the original cohort. Although this is an important strength of the study, the limited sample size and number of outcome events constrained the complexity of multivariable modeling. Therefore, despite adjustment for several clinically relevant baseline factors, some risk of model instability remains, and the adjusted estimates should be interpreted with caution. An additional limitation relates to the proportional hazards assumption in the multivariable Cox models, which was not fully satisfied and appeared to be violated primarily by pretreatment PNI. This suggests that the association of pretreatment PNI with PFS and OS may vary over time and may be more pronounced during the earlier post-infusion period. Accordingly, the reported hazard ratios should be interpreted as average effects over follow-up rather than strictly constant effects. Therefore, the multivariable findings should not be interpreted as establishing a definitive and time-invariant independent prognostic effect of pretreatment PNI. The 24-month restricted follow-up analysis was included as an exploratory sensitivity analysis to examine this early risk pattern, but it does not replace formal modeling of time-varying coefficients. Because of the limited sample size and number of events, we did not fit a more complex time-varying coefficient model in the present study. In addition, because 23 of the 87 treated patients were not included in the final analytic cohort, some degree of selection bias cannot be excluded, even though descriptive comparisons of the available baseline characteristics between included and excluded patients did not reveal obvious imbalances. As a result, the final analytic cohort may not fully represent the entire treated population. Another limitation is the histological heterogeneity of the study population. Although all patients had relapsed or refractory B-cell non-Hodgkin lymphoma and received the same TanCAR7 platform, different histological subtypes may have distinct biological behavior, treatment sensitivity, and survival patterns. Therefore, the present findings should be interpreted with additional caution across histological subgroups, particularly for indolent lymphoma, in which the subgroup size was small and the estimates are therefore less stable. Validation in larger and more diverse cohorts will be necessary. Second, toxicity data were not uniformly available for all CAR-T-related adverse events of interest. In particular, immune effector cell-associated neurotoxicity syndrome (ICANS) was not prospectively collected as a standardized variable in the original study dataset and therefore could not be evaluated in the present retrospective analysis. As a result, although we were able to explore the association between pretreatment PNI and cytokine release syndrome, we could not determine whether pretreatment PNI was also related to neurotoxicity risk. PNI was assessed at a single pretreatment time point only. Dynamic changes in immunonutritional status before and after infusion were not available. Moreover, other nutritional and body composition measures, including sarcopenia-related parameters, were not systematically collected and therefore could not be analyzed. Future prospective studies should therefore evaluate longitudinal PNI kinetics and incorporate additional nutritional and body composition measures, such as sarcopenia-related parameters, to better define the relationship between immunonutritional status and outcomes after CAR-T therapy. In addition, complementary measures of nutritional reserve and host fitness, such as CT-based sarcopenia assessment, skeletal muscle indices, or bioimpedance-derived parameters, were not systematically collected in this retrospective cohort and therefore could not be incorporated into the analysis. Finally, given the observational nature of the study, we cannot determine whether low PNI is directly involved in the biological mechanisms underlying poor outcome or whether it primarily reflects other adverse host-related conditions that were not fully captured in our dataset.

Despite these limitations, this study highlights a clinically relevant point. In the CAR-T setting, prognosis is determined not only by the lymphoma itself, but also by the condition of the patient who receives treatment. Pretreatment PNI is attractive because it is simple, inexpensive, and immediately available from routine testing. Although it is not sufficient to guide treatment selection on its own, it may help identify patients who need closer early monitoring and more individualized supportive care around CAR-T therapy.

In conclusion, pretreatment PNI was associated with response and survival in patients with relapsed or refractory B-cell non-Hodgkin lymphoma treated with TanCAR7. In this exploratory cohort, pretreatment PNI may provide additional prognostic information, particularly for early risk stratification after infusion, but the survival findings should be interpreted cautiously given the limited number of events and the apparent time-varying effect. Its potential clinical value appears to lie less in changing the CAR-T regimen and more in improving pretreatment assessment, early follow-up, and supportive care planning. Prospective studies are needed to confirm these findings and to determine whether interventions targeting nutritional and host fitness status can improve outcomes in patients with low PNI.

## 5. Conclusions

In patients with relapsed/refractory B-cell non-Hodgkin lymphoma treated with TanCAR7, pretreatment PNI was associated with response and survival. In multivariable analyses, pretreatment PNI remained associated with PFS and OS after adjustment for selected baseline factors, but these findings should be interpreted cautiously given the limited number of events and the apparent time-varying effect. Its prognostic value appeared to be most evident during the early period after infusion, suggesting a role in baseline risk stratification before CAR-T therapy. As a simple index derived from routine laboratory parameters, PNI may complement established clinical factors in pretreatment assessment. Prospective validation in larger cohorts is warranted.

## Figures and Tables

**Table 1:** Baseline characteristics according to pretreatment prognostic nutritional index group.

Characteristic	Total (*n* = 64)	Low PNI (<45) (*n* = 27)	High PNI (≥45) (*n* = 37)	*p* Value
**Age at CAR-T therapy, years**				
<60	54 (84.4%)	23 (85.2%)	31 (83.8%)	1.000
≥60	10 (15.6%)	4 (14.8%)	6 (16.2%)	
**Gender**				
Male	29 (45.3%)	11 (40.7%)	18 (48.6%)	0.615
Female	35 (54.7%)	16 (59.3%)	19 (51.4%)	
**BMI**				
<24	36 (56.2%)	16 (59.3%)	20 (54.1%)	0.800
≥24	28 (43.8%)	11 (40.7%)	17 (45.9%)	
**ECOG score**				
0–1	43 (67.2%)	16 (59.3%)	27 (73.0%)	0.289
≥2	21 (32.8%)	11 (40.7%)	10 (27.0%)	
**Primary diagnosis**				
CLL/SLL	1 (1.6%)	1 (3.7%)	0 (0.0%)	
DLBCL	42 (65.6%)	16 (59.3%)	26 (70.3%)	
FL	10 (15.6%)	4 (14.8%)	6 (16.2%)	
MALT	1 (1.6%)	1 (3.7%)	0 (0.0%)	
MCL	1 (1.6%)	0 (0.0%)	1 (2.7%)	
PMBCL	4 (6.2%)	1 (3.7%)	3 (8.1%)	
TFL	5 (7.8%)	4 (14.8%)	1 (2.7%)	
**Activity of lymphoma**				
Aggressive	51 (79.7%)	21 (77.8%)	30 (81.1%)	0.763
Indolent	13 (20.3%)	6 (22.2%)	7 (18.9%)	
**Primary refractory**				
Yes	39 (60.9%)	16 (59.3%)	23 (62.2%)	1.000
No	25 (39.1%)	11 (40.7%)	14 (37.8%)	
**Stage**				
Advanced	53 (82.8%)	25 (92.6%)	28 (75.7%)	0.100
Limited	11 (17.2%)	2 (7.4%)	9 (24.3%)	
**IPI score**				
<3	37 (57.8%)	11 (40.7%)	26 (70.3%)	0.023
≥3	27 (42.2%)	16 (59.3%)	11 (29.7%)	
**B symptom**				
Yes	25 (39.1%)	13 (48.1%)	12 (32.4%)	0.300
No	39 (60.9%)	14 (51.9%)	25 (67.6%)	
**Ki-67**				
<70%	28 (43.8%)	15 (55.6%)	13 (35.1%)	0.130
≥70%	36 (56.2%)	12 (44.4%)	24 (64.9%)	
**Extranodal lesions**				
Yes	45 (70.3%)	22 (81.5%)	23 (62.2%)	0.107
No	19 (29.7%)	5 (18.5%)	14 (37.8%)	
**Tumor burden (SPD)**				
<39.5 cm^2^ (median)	32 (50.0%)	8 (29.6%)	24 (64.9%)	0.011
≥39.5 cm^2^ (median)	32 (50.0%)	19 (70.4%)	13 (35.1%)	
**Lines of therapy before CAR-T**				
<3 (median)	19 (29.7%)	9 (33.3%)	10 (27.0%)	0.594
≥3 (median)	45 (70.3%)	18 (66.7%)	27 (73.0%)	
**LDH before CAR-T**				
>ULN	26 (40.6%)	14 (51.9%)	12 (32.4%)	0.132
Normal	38 (59.4%)	13 (48.1%)	25 (67.6%)	
**Albumin before CAR-T, g/L**				
<40.5	32 (50.0%)	23 (85.2%)	9 (24.3%)	<0.001
≥40.5	32 (50.0%)	4 (14.8%)	28 (75.7%)	
**Absolute lymphocyte count before CAR-T, ×10^9^/L**				
<0.30	29 (45.3%)	13 (48.1%)	16 (43.2%)	0.801
≥0.30	35 (54.7%)	14 (51.9%)	21 (56.8%)	
**Prior HSCT/ASCT**				
Yes	7 (10.9%)	3 (11.1%)	4 (10.8%)	1.000
No	57 (89.1%)	24 (88.9%)	33 (89.2%)	
**Prior radiotherapy**				
Yes	16 (25.0%)	8 (29.6%)	8 (21.6%)	0.563
No	48 (75.0%)	19 (70.4%)	29 (78.4%)	
**Prior anti-CD19 CAR-T**				
Yes	5 (7.8%)	1 (3.7%)	4 (10.8%)	0.387
No	59 (92.2%)	26 (96.3%)	33 (89.2%)	
**CAR-T cell dose**				
<3.8 × 10^6^/kg (median)	31 (48.4%)	13 (48.1%)	18 (48.6%)	1.000
≥3.8 × 10^6^/kg (median)	33 (51.6%)	14 (51.9%)	19 (51.4%)	

**Abbreviations:** BMI, body mass index; ; CLL, chronic lymphocytic leukemia; SLL, small lymphocytic lymphoma; IPI, International Prognostic Index; SPD, sum of the products of diameters; LDH, lactate dehydrogenase; ULN, upper limit of normal; HSCT, hematopoietic stem cell transplantation; ASCT, autologous stem cell transplantation; CAR, chimeric antigen receptor.

**Table 2:** Best overall response according to pretreatment prognostic nutritional index group.

Variable	Overall (*n* = 64)	High Group (*n* = 37)	Low Group (*n* = 27)	*p* Value
CR	44 (68.8%)	30 (81.1%)	14 (51.9%)	0.021
PR	6 (9.4%)	3 (8.1%)	3 (11.1%)	1.000
SD	4 (6.3%)	1 (2.7%)	3 (11.1%)	0.289
PD	10 (15.6%)	3 (8.1%)	7 (25.9%)	0.082
ORR	50 (78.1%)	33 (89.2%)	17 (63.0%)	0.024
DCR	54 (84.4%)	34 (91.9%)	20 (74.1%)	0.075

**Abbreviations:** CR, complete response; PR, partial response; SD, stable disease; PD, progressive disease; ORR, objective response rate; DCR, disease control rate; PNI, prognostic nutritional index.

**Table 3:** Descriptive summary of cumulative deaths at different follow-up time points according to pretreatment prognostic nutritional index group.

Time Point	Overall Deaths, *n*	Rate (%)	Low PNI (<45), *n*	High PNI (≥45), *n*	*p* Value
≤1 year	9	14.1	9	0	0.002
≤2 years	14	21.9	13	1	<0.001
≤3 years	21	32.8	13	8	0.033
≤5 years	26	40.6	13	13	0.316

**Table 4:** CRS outcomes according to pretreatment PNI category.

CRS Outcome	Total (*n* = 64)	Low PNI (<45) (*n* = 27)	High PNI (≥45) (*n* = 37)	Odds Ratio (95% CI)	*p* Value
Any CRS	56 (87.5%)	26 (96.3%)	30 (81.1%)	6.07 (0.70–52.61)	0.124
Grade 1	45 (70.3%)	18 (66.7%)	27 (73.0%)	—	—
Grade 2	6 (9.4%)	4 (14.8%)	2 (5.4%)	—	—
Grade 3	4 (6.3%)	3 (11.1%)	1 (2.7%)	—	—
Grade 4	1 (1.6%)	1 (3.7%)	0 (0.0%)	—	—
CRS grade ≥2	11 (17.2%)	8 (29.6%)	3 (8.1%)	4.77 (1.13–20.16)	0.042
CRS grade ≥3	5 (7.8%)	4 (14.8%)	1 (2.7%)	6.26 (0.66–59.58)	0.153

## Data Availability

The original contributions presented in the study are included in the article and Supplementary Material. Further inquiries can be directed to the corresponding authors.

## Supplementary Materials

The following supporting information can be downloaded at: https://www.mdpi.com/article/10.3390/nu18203425/s1, Table S1. Comparison of baseline characteristics between included and excluded patients; Table S2. Treatment response and survival outcomes according to three-category pretreatment prognostic nutritional index; Table S3. Subgroup analysis of treatment response according to pretreatment PNI by lymphoma subtype; Figure S1. Schoenfeld residual plots for pretreatment PNI in the multivariable Cox models for progression-free survival (A) and overall survival (B); Table S4. Sensitivity analyses restricted to the first 24 months of follow-up; Figure S2. Kaplan–Meier curves for overall survival (A) and progression-free survival (B) according to pretreatment prognostic nutritional index in the aggressive lymphoma subgroup.
