# Supplementary material for: Pretreatment Prognostic Nutritional Index Is Associated with Treatment Response and Survival in Patients with Relapsed or Refractory Non-Hodgkin Lymphoma Receiving CAR-T Cell Therapy
Source: Nutrients. 2026 Oct 19;18(20):3425. doi: 10.3390/nu18203425 (PMC13708103; doi:10.3390/nu18203425)
Supplement: Supplementary file 1 [file nutrients-18-03425-s001.zip › nutrients-4590064-SI.pdf]

Supplementary Table S1. Comparison of baseline characteristics between included and excluded patients.

Characteristic	Included (n=64)	Excluded (n=23)	P value
Age, years, mean ± SD	47.6 ± 12.4	47.3 ± 10.7	0.909
Age > 60 years, n (%)	10 (15.6)	3 (13.0)	1.000
Age ≤ 60 years, n (%)	54 (84.4)	20 (87.0)	
Sex			
Male	29 (45.3)	12 (52.2)	0.748
Female	35 (54.7)	11 (47.8)	
Histology, n (%)			1.000
Aggressive lymphoma	51 (79.7)	18 (78.3)	
Indolent lymphoma	13 (20.3)	5 (21.7)	
Primary refractory disease, n (%)	39 (60.9)	16 (69.6)	0.628
Tumor burden (SPD, cm^2), median (IQR)	39.5 (26.1-108.5)	65.0 (29.0-94.5)	0.689

Supplementary Table S2. Treatment response and survival outcomes according to three-category pretreatment prognostic nutritional index.

Pretreatment PNI category	CR, n (%)	ORR, n (%)	Median PFS, months (95% CI)	Median OS, months (95% CI)	1-year OS, % (95% CI)	2-year OS, % (95% CI)
PNI < 40	14 (66.7)	16 (76.2)	18.0 (6.2–49.6)	20.5 (8.2–69.6)	70.6 (42.5–98.7)	48.9 (3.0–94.7)
PNI 40–45	3 (42.9)	4 (57.1)	33.2 (3.4–83.9)	33.2 (3.4–83.9)	57.1 (0.0–100.0)	57.1 (0.0–100.0)
PNI > 45	32 (88.9)	34 (94.4)	NR	NR	100.0(100.0–100.0)	97.2 (91.7–100.0)
P value	0.013	0.021*	<0.001	<0.001		

Supplementary Table S3. Subgroup analysis of treatment response according to pretreatment PNI by lymphoma subtype.

Variable	Overall cohort	Aggressive lymphoma	Indolent lymphoma
Patients, n (%)			
Total n	64 (100.0)	51 (100.0)	13 (100.0)
Low PNI, n (%)	27 (42.2)	21 (41.2)	6 (46.2)
High PNI, n (%)	37 (57.8)	30 (58.8)	7 (53.8)
Treatment response, n (%)			
CR, Low PNI	17 (63.0)	12 (57.1)	5 (83.3)
CR, High PNI	32 (86.5)	26 (86.7)	6 (85.7)
CR p value	0.038	0.024	1
ORR, Low PNI	20 (74.1)	14 (66.7)	6 (100.0)
ORR, High PNI	34 (91.9)	27 (90.0)	7 (100.0)
ORR p value	0.081	0.064	1

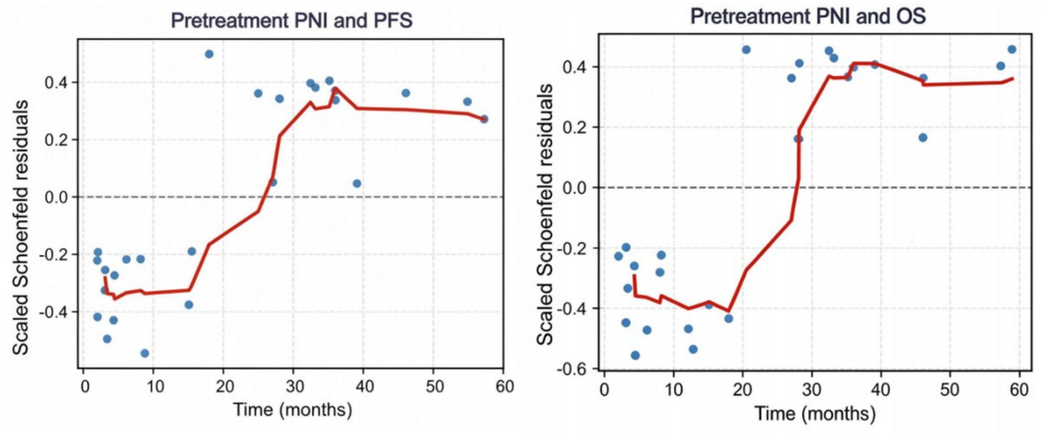

Supplementary Figure S1. Schoenfeld residual plots for pretreatment PNI in the multivariable Cox models for progression-free survival (A) and overall survival (B). Schoenfeld residual-based tests indicated a time-varying effect of pretreatment PNI in both models (PFS: $\rho = 0.666$, $p = 0.001$; OS: $\rho = 0.720$, $p < 0.001$).

Supplementary Table S4. Sensitivity analyses restricted to the first 24 months of follow-up.

Outcome	Events, low PNI	Events, high PNI	HR (95% CI)	P value
OS (0–24 months)	13	1	25.64 (3.34–196.73)	0.002
PFS (0–24 months)	13	1	25.76 (3.35–197.83)	0.002

Estimates should be interpreted cautiously because of the limited number of events within the restricted follow-up window.

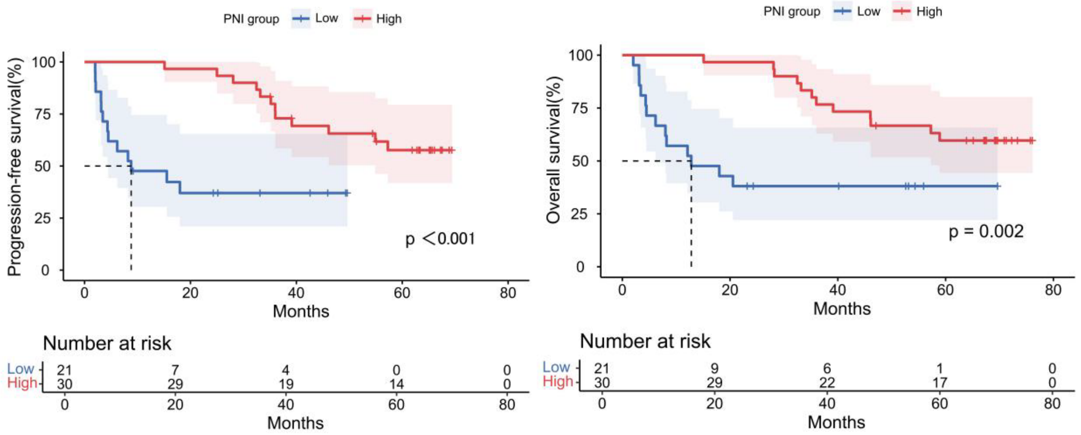

Supplementary Figure S2. Kaplan–Meier curves for overall survival (A) and progression-free survival (B) according to pretreatment prognostic nutritional index in the aggressive lymphoma subgroup.
